# Fresh Pork as Protein Source in the USDA Thrifty Food Plan 2021: A Modeling Analysis of Lowest-Cost Healthy Diets

**DOI:** 10.3390/nu15081897

**Published:** 2023-04-14

**Authors:** Romane Poinsot, Matthieu Maillot, Adam Drewnowski

**Affiliations:** 1 MS-Nutrition, 13005 Marseille, France; 2 Center for Public Health Nutrition, University of Washington, Seattle, WA 98195, USA

**Keywords:** Thrifty Food Plan 2021, USDA, national food prices, quadratic programming, meat, beef, pork, nutrient density, affordability

## Abstract

The USDA Thrifty Food Plan (TFP) is an estimate of a lowest-cost healthy diet that meets dietary guidelines while respecting existing eating habits. In the US, the TFP provides the basis for federal food assistance. Included in the TFP are protein foods from both animal and plant sources. The present goal was to explore the place of fresh pork among protein foods in the revised TFP 2021. Our analyses used the same databases and the same quadratic programming (QP) methods as had been used by the USDA to develop the TFP 2021. Dietary intakes came from the National Health and Nutrition Examination Survey (NHANES 2015-16); nutrient composition data came from the Food and Nutrient Database for Dietary Studies (FNDDS 2015-16), and national food prices came from the 2021 TFP report. Amounts and prices were for foods as consumed. Our QP Model 1 used USDA modeling categories to replicate the TFP 2021. The non-poultry meat category was then separated into pork and beef. Model 2 examined whether the TFP 2021 algorithm would select pork or beef. Model 3 sought the lowest cost healthy diet, the same as the TFP 2021. Model 4 replaced beef and poultry with pork; whereas Model 5 replaced pork and poultry with beef. Weekly costs were calculated for a family of four and eight age-gender groups. All models met the nutrient requirements. The market basket cost for a family of four in Model 1 was USD 189.88, compared to the purchase price of USD 192.84 in the TFP 2021. In Model 2, fresh pork was selected preferentially over beef. The lowest-cost healthy food plan in Model 3 increased fresh pork to 3.4 lbs/week. Replacing beef and poultry with pork in Model 4 led to a modest decrease in the weekly cost. Replacing pork and poultry with beef in Model 5 led to a major increase in the weekly cost. We conclude, based on TFP-analogous modeling, that fresh pork is the preferred meat source, providing high-quality protein at a low cost. QP methods, as used in the TFP 2021, are a valuable tool for designing food plans that are affordable, acceptable, and nutrient-rich.

## 1. Introduction

In 2021, the US Department of Agriculture (USDA) conducted an evidence-driven re-evaluation of the Thrifty Food Plan (TFP). The USDA has four food plans that estimate the cost of a healthy diet at different price points—the Thrifty, Low-Cost, Moderate-Cost, and Liberal Food Plans [1]. Of these, the TFP is the lowest cost. The TFP plays a critical role in calculating the amount of food assistance received in the US. By law, the estimated cost of the TFP is the basis for maximum benefits in the Supplemental Nutrition Assistance Program (SNAP) that begin during the following federal fiscal year [2,3].

The TFP is described as the lowest-cost healthy diet that meets nutrient needs, follows dietary guidance, and respects the population’s eating habits [2]. Estimating the cost of a nutritious, budget-conscious diet is a priority for the USDA [1]. Beginning with the TFP 1975, all TFP updates in the following 45 years had been cost-neutral [2], which gave rise to concerns that the inflation of food prices made healthy diets no longer affordable to the average consumer [4,5]. In addition, many households depleted food assistance benefits before the end of the month, increasing the risk of food insecurity [6]. The 2018 Farm Bill required the USDA to re-evaluate the TFP repeatedly using recent dietary guidance, current food prices, and current food patterns and food composition data [7].

The revised TFP 2021 was developed by the USDA Center for Nutrition Policy and Promotion (CNPP) using dietary intakes from the National Health and Nutrition Examination Survey (NHANES) [8], national food prices [9], and quadratic programming optimization methods [2]. No longer required to be cost-neutral, the TFP 2021 has provided updated cost estimates for a lowest-cost healthy diet [2]. Maximum benefits were increased by 21% [2], with average benefits rising by less than USD 2 per person per day [10]. The TFP 2021 cost for a reference family of four was now estimated at USD 192.84 per week [1].

The TFP 2021 allocates a percentage of the weekly budget (USD 47.45 or 24.61% cost share) to protein foods, defined by the USDA as meats and poultry, seafood, eggs, nuts, and soy products [2]. In the TFP 2021, meats were assigned into categories based on price and were divided into lower and higher nutrient-dense items [2]. The June 2021 food prices used in the TFP analyses were higher for meat than for poultry, thereby giving poultry a greater share of the protein foods in the TFP 2021 market basket [2]. Selections within the seafood category were likewise assumed to have lower prices [2].

In the TFP 2021, fresh beef and pork were combined into a single modeling category referred to as non-poultry meat (called “meat” in the TFP 2021 report). However, pork and beef can vary considerably in price. While multiple meat sources can satisfy nutrient needs, leading to practical and healthy diets, pork might have a price advantage.

Our goal was to replicate the TFP 2021 calculations as closely as possible while separating fresh pork from beef. The modeling category described as higher-nutrient density meats included numerous cuts of fresh pork: pork chop (baked, broiled, stewed, or fried), pork roast, and pork steak/cutlet [2]. Our quadratic programming (QP) models served to estimate the amounts of pork in the TFP 2021 and searched for the lowest-cost healthy diets with pork as the only source of meat. We used the same publicly available databases and modeling categories listed in the supplemental TFP 2021 data files [2,11], along with the 2021 national food prices. Our objective was to assess the nutritional value and affordability of a TFP that featured fresh pork as the principal meat source.

## 2. Materials and Methods

**Figure 1:**
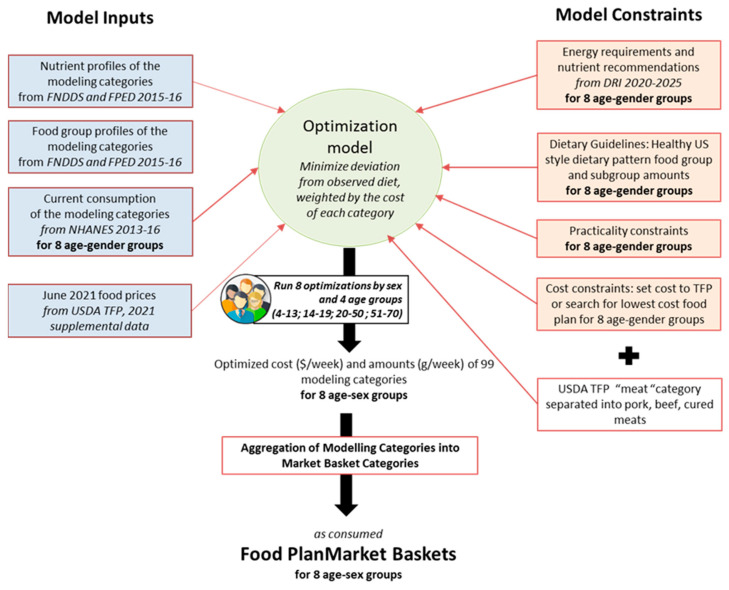
A schema showing the methods used to replicate the USDA TFP 2021 analyses.

The present methods closely followed the published TFP 2021 documentation [2]. Quadratic programming models require input data, a set of nutritional, social, or cost constraints, and an objective function [11,12]. In the TFP 2021 and the present models, input data came from the Dietary Guidelines for Americans [13] and the foods and modeling categories came from the USDA Food and Nutrient Database for Dietary Studies (FNDDS 2015-16) [14]. National food prices for over 3000 foods came from the USDA TFP 2021 supplemental data files [2]. The constraints ensured that the modeled food plans met energy requirements and nutrient recommendations for each population subgroup. The constraints ensured further that the generated food plans followed the USDA Healthy US-Style Food Pattern [15], did not deviate too much from observed dietary habits, and generated the lowest-cost healthy food plans. The cost constraint was applied progressively in USD 0.01 decrements until no mathematical solution was obtained. The five models generated optimized food plans for 99 modeling categories (amounts and cost). The procedures are shown in Figure 1.

The two phases of the process, each with multiple steps, tracked the CNPP methodology [2]. Phase one prepared the data sources, adapted the USDA modeling categories, and established the inputs and constraints. Phase two ran five QP optimization models to create five food plans for each of eight different age-gender groups.

There were some small differences in procedures. Our five QP models generated healthy TFP market baskets for 8 separate age-gender groups, as opposed to 15 [2]. Our reference family of four was composed of two adults aged 31–50 y and two children aged 4–13 y whereas the reference family of four in TFP 2021 consisted of two adults aged 31–50 y, one child 6–8 y, and one child 9–11 y. Whereas the TFP 2021 food amounts were presented “as purchased”, our QP analyses were for foods as consumed.

### 2.1. Input Data

#### 2.1.1. Separating Pork from Beef

Individual foods and beverages (i.e., “items”) in the FNDDS 2015-16 were identified by an 8-digit WWEIA food code [16]. The USDA TFP 2021 supplemental data [2] listed assignments of all FNDDS items into one of the 65 initial categories. Those TFP categories were largely based on What We Eat in America (WWEIA) 4-digit codes [16], albeit with some custom modifications by the CNPP. Selected food and beverage categories, including grains, fruit, dairy, protein foods (including meats), popcorn, and beverages, were split into subcategories of “higher” or “lower” nutrient density, largely determined by sodium, saturated fat, and sugar content. Inclusion criteria for the higher nutrient density categories are all detailed in the USDA TFP 2021 report Appendix 1 Table 1 [2].

In our coding scheme, pork items and beef items that were included in “meats, higher nutrient density” were separated into “pork, higher nutrient density” and “beef, higher nutrient density”. Similarly, our TFP analyses separated the category of “meats, lower nutrient density” into “pork, lower nutrient density” and “beef, lower nutrient density”. The distinction was largely based on saturated fat content below or above 4.5 g/100 g, as used by the CNPP [2]. This increased the number of TFP initial categories from the original 65 to 67. Aggregating initial categories that were separated according to nutrient density yielded a total of 46 so-called “combined” categories.

#### 2.1.2. Recreation of Modeling Categories Based on Food Prices

National food prices for 3072 foods and beverages were obtained from the USDA supplemental data files for the 2021 TFP [2]. The prices came from the 2015–2016 Purchase to Plate Price Tool [2] directly from retailers and were adjusted for inflation to June 2021 by the CNPP. Price outliers were excluded—items such as lobster or lamb were not included in the calculations of modeling category prices. The TFP 2021 created higher and lower price categories, based on the 35th percentile cut-point, for 30 out of 65 modeling categories. Food and beverage choices of higher-income households (>350% of federal poverty) were excluded from the weighted average modeling category price.

The 2015–2016 Purchase to Plate Price Tool provides the mean national retail prices for 3231 FNDDS food codes, i.e., for almost 97% of food and beverages reported in the What We Eat in America (WWEIA) studies [2]. The WWEIA study is the dietary intake component of the 2015-16 National Health and Nutrition Examination Survey (NHANES 2015-16). In developing the TFP 2021 [2], the USDA excluded from analysis 159 food codes (e.g., infant formula and food codes with small sample sizes).

Following the CNPP procedures, inflation-adjusted food prices were merged with the FNDDS nutrient composition data, and mean weighted prices were then calculated for each TFP food category and combined category, as described above. Missing prices and price outliers were excluded from the calculation of the weighted mean or, in other words, had a weight of zero. Outliers were defined as items with a price of more than 1.5 interquartile ranges above the first quartile of each TFP 2021 category and subcategory.

The distribution of weighted prices was then used to recreate the 99 modeling categories that were to be used as input variables in the optimization model (e.g., Section 2.2). Foods and beverages with prices at or below the 35th percentile of prices for the category were defined as “lower cost” items, whereas foods and beverages with prices above the 35th percentile were defined as “higher cost” items. These cut points were the same as those used in the 2021 TFP. One aim of the TFP 2021 was to create categories reflecting “thrifty” food choices, based on nutrient density relative to cost. One of those categories was red meat (i.e., fresh pork and beef but not lamb), shortened to “meat”. In the present analyses, beef and pork items were separated and assigned to lower-cost and higher-cost groups. In the present analyses, the higher-cost versus lower-cost distinction was thus applied to 32 out of the 67 initial categories (Supplemental Table S1).

#### 2.1.3. Weighted Nutrient Profiles and Food Pattern Components in the Modeling Categories

Mean weighted nutrient profiles were calculated for each modeling category. In the TFP 2021, the weights of foods and beverages were for items sourced from stores by individuals ages > 1 y with a poverty-to-income ratio > 3.5 on the first-day dietary recall in NHANES 2015–2016 [8]. The same method was used to estimate the mean amounts consumed. The modeling categories were linked to USDA Food and Nutrient Database for Dietary Studies (FNDDS) 2015–2016 [14] and to the USDA Food Patterns Equivalents Database (FPED) 2015-16 [17]. The FNDDS provides energy and nutrient values per 100 g, whereas the FPED converts individual foods and beverages into the 37 food-pattern components (e.g., fruits, vegetables, and dairy) that make up the USDA Healthy Food Patterns and are used to calculate HEI-2015 values. The FPED also lists added sugars.

#### 2.1.4. Current Food and Beverage Consumption Patterns

Food patterns optimized by linear or quadratic programming models ought to differ as little as possible from the existing eating habits [2]. The TFP 2021 quadratic programming model took into account nutrient adequacy, food prices, dietary guidance, and what Americans normally eat. The present estimates of the US population’s eating habits came from the first-day dietary recalls in two cycles of NHANES: 2013–14 and 2015–16. Average consumption patterns were calculated for males and females in age groups defined by ages: 4–13 y, 14–19 y, 20–50 y, and 51–70 y, that is, for eight age-gender groups in all. The TFP 2021 stratified the population into 15 groups by age and gender, from age 1 y to age 74 y. Our preference was to limit the age range from 4 y to 70 y.

The present goal was to create high-quality (i.e., nutrient-dense) food patterns at an affordable cost. The HEI-2015 score, a measure of compliance with Dietary Guidelines for Americans [18] was first calculated for each NHANES participant. An unweighted median HEI-2015 score was then estimated for males and females in each age group. For TFP modeling purposes, we only included NHANES participants with HEI-2015 scores above the group median. This was to ensure that nutrient-dense food items were included in the optimization model. The more nutrient-dense foods tend to be consumed by individuals with higher HEI-2015 scores. Current consumption patterns were expressed as quantities of combined categories whose average cost was already calculated (e.g., Section 2.1.2). Following TFP 2021 protocols that adjusted for plate waste and/or foods that may go uneaten before they spoil, a food waste adjustment factor of +5% was applied to the average consumption of each of the modeling categories for the eight age-gender groups.

#### 2.1.5. Energy, Nutrients, and Dietary Recommendations

Food plans developed by quadratic programming need to satisfy minimum energy and nutrient requirements, stay below maximum recommended values for nutrients of public health concern, and follow dietary guidelines for specific food groups or subgroups. The USDA Healthy U.S.-Style Dietary Pattern [15] is the recognized standard.

Consistent with the TFP 2021, the present analyses used nutrient standards based on the Dietary Reference Intakes (DRI) issued by the National Academies of Sciences, Engineering, and Medicine (NASEM) and on the most recent issue of the Dietary Guidelines for Americans, 2020–2025. The values, taken from the TFP 2021 report, were adapted to the age groups used in the present modeling studies. The lower and upper bounds of the dietary constraints for the present 4–13 y age group corresponded to the average recommendation for 4–5 y, 6–8 y, 9–11 y, and 12–13 y age ranges, weighted by the size of each age group in the modified NHANES 2013–2016 data (e.g., Section 2.1.3). They were also adjusted by five percent for food waste [2].

### 2.2. Quadratic Programming Models for Food Plan Optimization

An optimization model is defined by a set of input variables, a list of constraints, and an objective function that needs to be optimized (either minimized or maximized) [12]. The constraints in food plan optimization modeling generally correspond to nutrient requirements and the recommended distribution of food groups as set forth in agency standards and dietary guidelines. For the present analyses, nutrient standards came from the National Academies (NASEM) [19], whereas the recommended amounts of foods and beverages came from the Healthy U.S.-Style Dietary Pattern [15].

#### 2.2.1. Input Variables

The input variables were the quantities $x_{i}$ of each modeling category *i*, *i* = 1, …, 99. Modeling categories that describe the same food or beverage but declined into low-cost and high-cost and into low nutrient density and high nutrient density (Supplemental Table S1) were assigned to a combined category *j*, *j* = 1, …, 46.

#### 2.2.2. Objective Function

The objective function of the current optimization models was a quadratic function that minimized the overall distance between the quantity $x_{j}\;$of the combined category *j*, and the average consumption $c_{j}$, weighted by the expenditure shares of each combined category. This is given by Equation (1):

(1) $$\min\overset{46}{\underset{j=1}{\sum }}\beta_{j}{\left(x_{j}-c_{j}\right)}^{2}$$

$$\beta_{j}=\frac{p_{j}c_{j}}{\sum_{j=1}^{46}p_{j}c_{j}}$$

where $p_{j}\;$is the mean national price and $c_{j}\;$is the average amount of observed intake of combined category j.

#### 2.2.3. Nutritional and Food Group Constraints

Food plans generated by the present model had to meet the same nutrient and energy recommendations as the TFP 2021. Those came from DRI values established by NASEM. The first QP equation constraint (Equation (2)) is given by:

(2) $$D_{n}^{LB}\leq \overset{99}{\underset{i=1}{\sum }}x_{i}d_{i,n}\leq D_{n}^{UB}\;$$

where $d_{i,n}$ is the nutrient content per gram, $D_{n}^{LB}$ is the minimum daily recommendation, and $D_{n}^{UB}\;$is the maximum daily recommendation for nutrient *n* in the modeling category *i* for each age-gender group.

The recommendations needing to be met were for energy, protein, carbohydrates, fiber, added sugars, total lipid, saturated fatty acids, 18:2 linoleic acid, 18:3 linolenic acid, calcium, copper, iron, magnesium, phosphorus, potassium, sodium, zinc, vitamin A, thiamin, riboflavin, niacin, vitamin B6, folate, folic acid, vitamin B12, vitamin E, vitamin K, and choline. Vitamin D was not included because it is difficult to achieve the recommended intake of this nutrient through food sources alone.

In addition to meeting nutrient requirements, the optimized food plan must provide those food groups, subgroups, and other dietary components that make up healthy dietary patterns. Typically, acceptable ranges with upper and lower bounds are provided. The second QP equation (Equation (3)) is given by:

(3) $$F_{p}^{LB}\leq \overset{99}{\underset{i=1}{\sum }}x_{i}f_{i,p}\leq F_{p}^{UB}$$

where $f_{i,p}$ is the amount per gram, $F_{p}^{LB}$ is the lower bound, and $F_{p}^{UB}$ is the upper bound of the FPED food groups or subgroups *p* in the modeling category *i* for each age-sex group.

The food groups and subgroups in the present model were for the most part food pattern components of the Healthy U.S.-Style Dietary Pattern. In the FPED datafile, those were vegetables (group and subgroups), fruits, grains, dairy, protein foods, meats-poultry-eggs, seafood, nuts-seeds-soy, and oils. For those food groups, the $F_{p}^{LB}$ was the recommended amount in the Dietary Guidelines for Americans, 2020–2025 and the $F_{p}^{UB}$ was the 95th percentile of reported dietary intake [20]. In those cases where the 95th percentile of reported dietary intake was below the recommended amount, $F_{p}^{UB}$ corresponded to the recommended amount increased by 10% [2]. For aggregated food groups (i.e., meat, poultry, and eggs; nuts, seeds, and soy), that did not directly correspond to the Healthy U.S.-Style Dietary Pattern component, the $F_{p}^{LB}$ lower bound was the 25th percentile of the reported dietary intake and the $F_{p}^{UB}$ upper bound was the 95th percentile (or 75th percentile for eggs) of the reported dietary intake.

Further constraints followed the Dietary Guidelines for Americans [13]. One-half or more of the fruit group needed to come from the whole fruit sub-group (Equation (4a)) and one-half or more of the total grains needed to come from whole grains (Equation (4b)). In addition, one-half or more of the total amount of dairy needed to come from the higher nutrient-density milk and yogurt category (Equation (4c)). These food group constraints were addressed by Equations (4a)–(4c).

(4a) $$\overset{99}{\underset{i=1}{\sum }}x_{i}f_{i,\;p=whole\;fruit}\geq 0.5\;\overset{99}{\underset{i=1}{\sum }}x_{i}f_{i,\;p=total\;fruit}\;$$

(4b) $$\overset{99}{\underset{i=1}{\sum }}x_{i}f_{i,\;p=whole\;grains}\geq 0.5\;\overset{99}{\underset{i=1}{\sum }}x_{i}f_{i,\;p=total\;grains}\;$$

(4c) $$x_{i=milk\;and\;yogurt,\;hnd}\geq x_{i=milk\;and\;yogurt,lnd}\;$$

Combined categories that did not match a Healthy US Style Dietary Pattern component were limited to no more than two standard deviations above the average amount of the observed intake (Equation (5)). Those were milk substitutes, nutritional beverages, and smoothies; fruit drinks; soda; biscuits, muffins, and quick breads; condiments and sauces; mixed dishes; butter and animal fats; margarine, oils, cream, and cream substitutes; potatoes; sweet bakery products; snack bars; candy; tortilla, corn, and other chips; crackers; other desserts; pretzels/snack mixes; popcorn; and sugar and sugar substitutes. This constraint was addressed by Equation (5).

(5) $$for\;j\notin \left\{\mathrm{Healthy}\;\mathrm{US}\;\mathrm{Style}\;\mathrm{Dietary}\;\mathrm{Pattern}\;\mathrm{component}\right\},\;0\leq x_{j}\leq c_{j}-2*sd_{j}\;$$

where $c_{j}$ is the average amount of observed intake and $sd_{j}$ the standard deviation of observed intake of combined category j.

The TFP 2021 addressed selected beverages. Most US adults drink coffee or tea. The optimized food plan for adults ≥ 20 y contained a minimum of 1 cup per day (=240 g) of coffee or tea. The plan for children and teenagers ages 19 and younger did not contain any coffee or tea (Equation (6)). The Equation (6) is:

(6) $$x_{j=coffee\;and\;tea}\geq \;240\;\mathrm{for}\;\mathrm{adults}\;\mathrm{and}\;x_{j=coffee\;and\;tea}=0\;\mathrm{for}\;\mathrm{ages}\geq 19\;y\;$$

Finally, the 2021 TFP limited the total number of calories associated with the breakfast eating occasion to 23 percent. Equation (7) is given by:

(7) $$\overset{99}{\underset{i=1}{\sum }}x_{i}breakfast\;energy_{i}\leq 0.23\;\overset{99}{\underset{i=1}{\sum }}x_{i}energy_{i}\;$$

where $breakfast\;energy_{i}$ is the number of calories in the modeling category *i* that is associated with the breakfast eating occasion and $energy_{i}\;$is the total number of calories in the modeling category *i*.

#### 2.2.4. Cost Constraints

Cost constraint was imposed depending on the model, as defined in Table 1. For Model 1 (a replica of the TFP 2021), the lowest cost was determined by decreasing or increasing the observed diet cost by USD 0.01. For Model 2, designed to estimate the contribution of beef and pork separately, the cost constraint was set to the cost of the M1 model, that is TFP 2021. For all other models (M3, M4, and M5) the lowest cost was searched for by lowering the cost of the observed diet by USD 0.01. The cost constraint is given by Equation (8):

(8) $$\overset{99}{\underset{i=1}{\sum }}x_{i}p_{i}\leq C_{obs}-C\;\mathrm{for}\;M1,\;M3,\;M4,\;M5\;\mathrm{or}\;\overset{99}{\underset{i=1}{\sum }}x_{i}p_{i}=C_{TFP}\;\mathrm{for}\;M2$$

$$C_{obs}=\overset{46}{\underset{j=1}{\sum }}c_{j}p_{j}\;\mathrm{and}\;C\;\in \left\{0,\;0.01,\;0.02,\;0.03,\;\ldots \right\}$$

where $c_{j}$ is the average amount of observed intake, $p_{i}$ is the national average price of modeling category i, $C_{obs}$ is the observed cost of the diet, C is the cost decrease, and $C_{TFP}$ is the cost of the 2021 TFP-optimized diet.

### 2.3. Five QP Optimization Models

Five different QP optimization models were developed for each of the eight age-gender groups. Model 1 was intended to replicate the USDA TFP 2021 as closely as possible, using the same modeling categories and cost constraints. The minimal cost was determined by imposing the cost constraint in USD 0.01 decrements or increments from the observed cost (Equation (8)) until no solution was found. This model generated the lowest-cost nutrient-adequate food plan using the single category “meat”.

Model 2 separated the meat category into fresh pork and fresh beef, adding four new modeling categories. The sum of beef and pork in Model 2 was set to be the same as the amount of the “meat” category in Model 1, which corresponded to the TFP 2021. The amounts of other protein foods were determined by the model. The cost was set to be the same as the Model 1. The question was whether Model 2 would choose only pork to arrive at the lowest-cost food plan.

Model 3 also separated fresh pork from beef and searched for the lowest-cost solution, following the USDA TFP approach. The amounts of pork, beef, and other protein foods in Model 3 were determined by the model.

Model 4 replaced red meat and poultry (both set to zero) with fresh pork. The amounts of other protein foods were defined by the model. The minimal cost was determined by imposing the cost constraint in USD 0.01 decrements or increments (Equation (8)) until no solution was found. The goal was to determine the lowest cost of healthy diets where pork was the only source of meat.

Model 5 replaced pork and poultry (both set to zero) with beef. The amounts of other protein foods were defined by optimization. The minimal cost was determined by imposing the cost constraint in USD 0.01 decrements or increments (Equation (8)) until no solution was found. The goal was to estimate the lowest cost of healthy diets where beef was the only source of animal meat.

### 2.4. Analysis

Modeling categories were combined into “as consumed” market basket categories aggregated as shown in Supplemental Table S1. In the USDA market basket categories, meats and cured meat modeling categories were aggregated into one category, also called “meat”. In our analysis, we decided to separate cured meat from beef and pork in the ‘as-consumed” market basket categories. Optimized amounts were converted from daily to weekly quantities by multiplying them by seven. Weekly costs were estimated for the entire diet and food categories “as consumed”. Weekly costs and totals, determined by category, were also estimated for a family of four persons consisting of one male 20–50 y, one female 20–50 y, one male 4–13 y, and one female 4–13 y. For comparison, the USDA reference family of four is composed of an adult male and female and two children aged 6–8 y and 9–11 y.

## 3. Results

### 3.1. TFP Weekly Cost for a Family of Four: USDA 2021 TFP and QP Model 1

Our QP Model 1 followed the TFP 2021 model exactly. The estimated costs and the amounts “as consumed” were the same as determined by the CNPP for the same age groups. The meat category included both pork and beef and the QP optimization searched for the lowest-cost food plan, the same as the TFP. First, the weekly cost of the Model 1 market basket for our reference family of four was USD 189.88, as compared to the TFP 2021 estimate of USD 192.84. As noted above, the TFP report listed amounts as purchased, whereas our calculations dealt with the foods as consumed. For some food categories, notably vegetables and fruit, there was a difference in yield because of preparation and waste. As a result, food amounts as consumed are generally less than food amounts as purchased. Food prices are calculated by the USDA per 100 g of edible portions, already corrected for preparation and waste [2].

**Figure 2:**
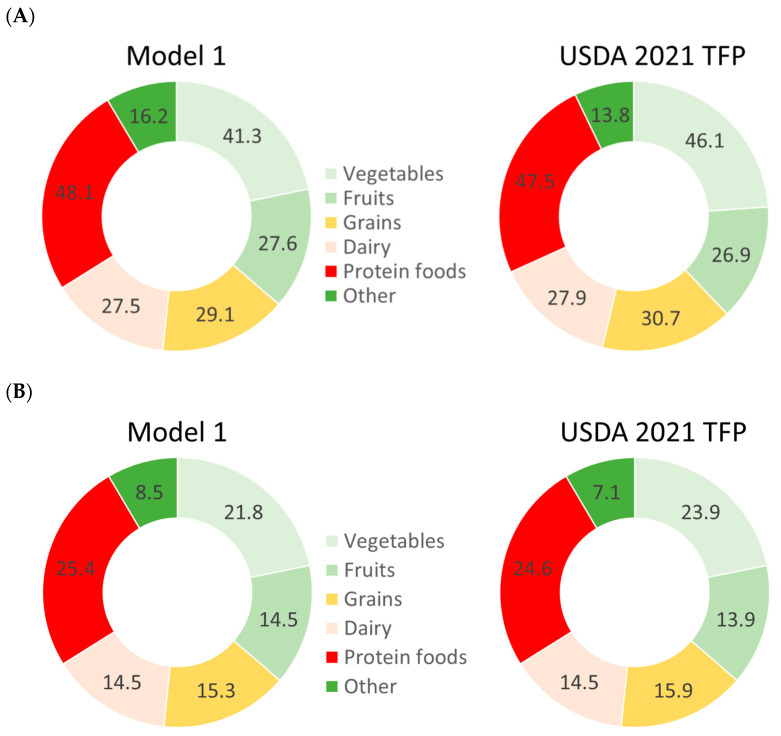
Estimated TFP cost in USD/week (**A**) and estimated cost share in % (**B**) by food category for a family of four.

Figure 2 shows the cost distribution of the USDA TFP and Model 1 by category and the percentage cost share by category. There was a close correspondence between the USDA 2021 TFP and our Model 1.

**Figure 3:**
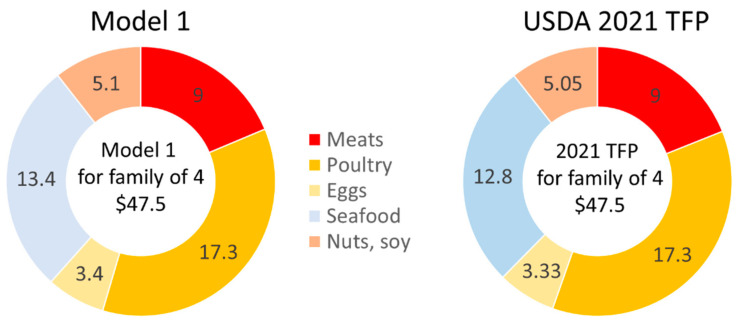
Estimated TFP weekly cost (USD/week) by protein food category for a family of four.

The weekly cost of the protein foods in our QP Model 1 was USD 48.13 (or 25.3% cost share) as compared to the TFP 2021 estimate of USD 47.45 (or 24.6% cost share) (Figure 2). The cost of meats in QP Model 1 was USD 9.0/week compared to USD 8.96 in the TFP 2021 (Figure 3).

Model 1 estimated the number of protein foods at 14.18 lbs/week for a family of four, compared to the TFP 2021 estimate of 16.18 lbs/week. The amount of meats consumed was 2.05 lbs/week, compared to 2.26 lbs/week in the TFP 2021. The cost shares for different food categories were much the same as those reported in the USDA TFP 2021.

### 3.2. The Place of Pork in Optimized TFP Models

Our QP Model 2 was designed to test whether the TFP would preferentially select pork or beef. The USDA single “meat” modeling category was now divided into pork and beef. The total weekly cost of the TFP was set to be the same as in Model 1, which is USD 189.87. The weekly cost of protein foods was USD 46.68, including USD 6.90 for meats. For the lowest-cost healthy food plan, Model 2 selected only fresh pork (but no beef) and increased the amount of poultry. The amounts of eggs, seafood, nuts, and soy remained the same as before.

**Figure 4:**
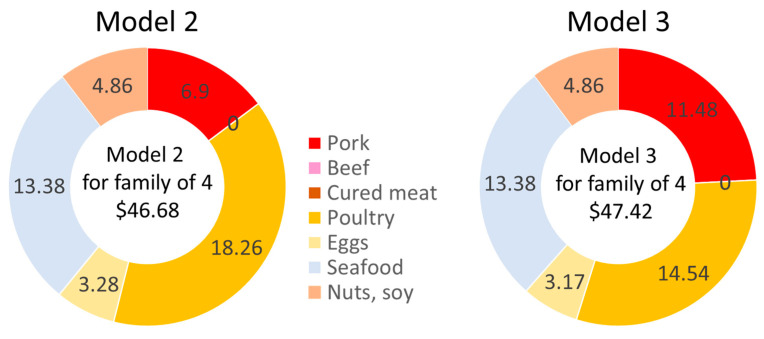
Estimated costs (USD/week) of models 2 and 3 by protein food category.

Our QP Model 3 continued to separate fresh pork from beef and searched for the lowest-cost nutrient adequate food plan. The weekly cost of the market basket as determined by Model 3 was USD 187.81, very close to the reported optimized TFP 2021 values. The weekly cost of the protein foods was USD 47.42, including USD 11.48 for fresh pork (Figure 4). In the protein foods meat category, Model 3 also selected only fresh pork (no beef or cured meats), increasing the amount of pork from 2.05 lbs/week to 3.40 lbs/week and reducing the amount of poultry to 4.52 lbs/week. The amounts of eggs, seafood, nuts, seeds, and soy remained the same. Model 3 showed that a nutrient-adequate lowest-cost diet can be obtained using pork as the only source of non-poultry meat.

As shown in Figure 4, when the weekly cost was pegged to the TFP 2021, Model 2 selected only fresh pork and no beef or cured meat. When the QP Model 3 searched for the lowest-cost healthy diet, the market basket contained more pork, less chicken, and no cured meats or beef.

### 3.3. A Direct Comparison of Food Plans with Only Pork or Beef

**Figure 5:**
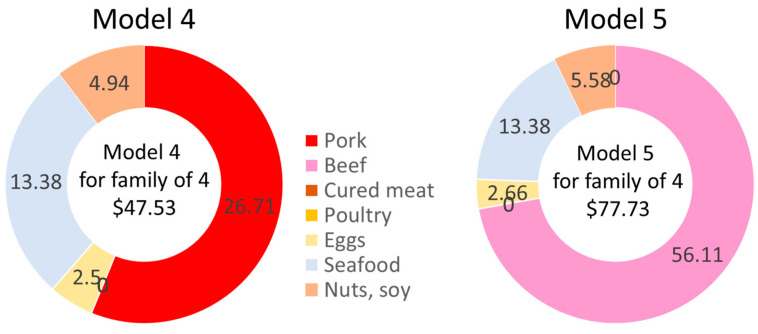
Estimated costs in USD/week of Model 4 (pork only) and Model 5 (beef only) by protein food category.

Our QP Models 4 and 5 searched for the lowest-cost healthy diets with fresh pork replacing both beef and poultry (but not seafood). Model 4 set beef and poultry amounts to zero and searched for the lowest cost nutrient-adequate food plan, with pork as the only source of all meat (other than seafood). The weekly cost of the food plan as determined by Model 4 was USD 188.57, lower but very close to the USDA TFP values (Figure 5). The weekly cost of the protein foods was USD 47.53, with USD 26.71 going to fresh pork. In this model, pork replaced both beef and poultry. The amounts of eggs, seafood, nuts, seeds, and soy remained the same as before.

Our QP Model 5 set pork and poultry amounts to zero and searched for the lowest cost nutrient-adequate food plan, with beef as the only source of all meat (other than seafood). The weekly cost of the Model 5 food plan was USD 220.58/week, with USD 77.73 going to protein foods (Figure 5). The cost of the healthy beef-only diet was substantially above the USDA TFP 2021 and was also higher than the estimated cost of the present QP models 1 through 4. Poultry was set as zero and the amounts of eggs, seafood, nuts, seeds, and soy remained the same.

These data are also summarized in Table 2 and Table 3 below. Table 2 shows, for each of the QP models, quantities of foods as consumed (lbs/wk) by food category as well as the costs of each food plan (USD/wk) by category for a family of four.

Table 3 shows, for each of the QP models, quantities of foods as consumed (lbs/wk) by protein food category as well as the costs of each food plan (USD/wk) by category. The data are calculated for a family of four.

### 3.4. The Place of Pork in Low-Cost Healthy Diets for Adults Aged 20–50 y

**Figure 6:**
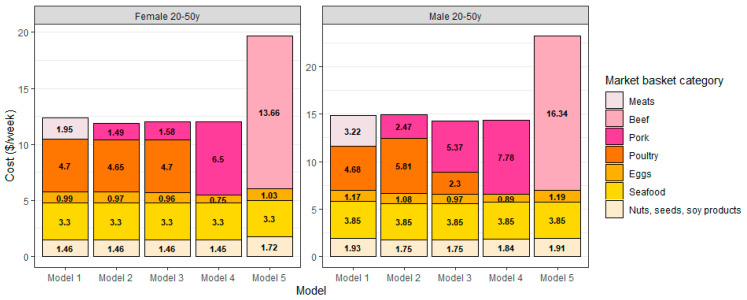
Protein foods costs per week (other age-gender groups in Supplemental Table S2).

Figure 6 shows the place of pork in each of the five QP food plans. The data are presented separately for women (A) and men (B) aged 20–50 y. Shown are the weekly costs in USD/week for protein foods: meat, poultry, eggs, seafood and nuts, and soy. Cured meat costs were not shown because they were always equal to 0 USD/week. Model 1 combined pork and beef into a single category and Model 2 separated pork from beef. Here, Model 2 preferentially selected pork in the place of beef to arrive at the healthy food plan at the TFP 2021 cost. Model 3 showed that pork was the only non-poultry meat in the lowest-cost food plan. Model 4 showed that pork could substitute for beef and poultry without an increase in cost. Model 5 showed that replacing pork and poultry with beef led to a substantial increase in weekly cost. All models generated healthy food patterns, meeting energy and nutrient requirements and the distribution of food groups following the Dietary Guidelines for Americans.

### 3.5. Optimized Food Plans Calculated for Eight Age-Gender Groups

Table 4 shows that the overall cost of each food plan USD/week and the costs of the protein foods closely tracked the USDA TFP 2021 estimates for the same age-gender groups. However, whereas the USDA calculated costs for children aged 4–5 y, 6–8 y, and 9–11 y and for males and females aged 12–13 y, our analyses only had males and females aged 4–13 y.

Our prices for foods as consumed in Model 1 were exactly the same as the TFP 2021 estimates. There was also a substantial agreement between Models 2 through 4 and the TFP 2021 estimates of the lowest-cost healthy diets. Only Model 5, where beef replaced both red meat and poultry, was associated with a much higher weekly cost. One group for whom the costs of a beef-only diet were particularly elevated was young males aged 14–19 y. The modeled QP food plans do not depart too much from the population group’s habitual diet.

### 3.6. Optimized Food Plans by Age-Gender Group and Food Category

**Figure 7:**
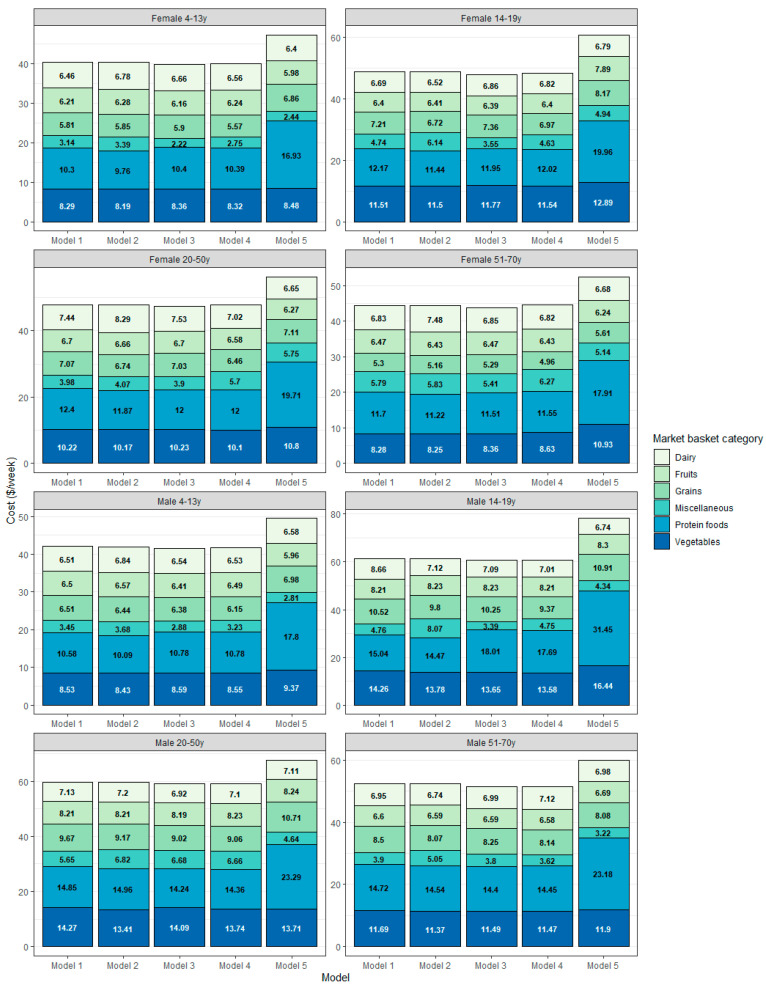
Food plan costs in USD/week by age-gender type, model type, and food category.

Figure 7 shows, for each age-gender group, and each QP model, the cost of foods as consumed (USD/week) by food category.

## 4. Discussion

The USDA food plans provide an optimization structure for a healthy, nutrient-adequate diet at different price points [1]. The estimated cost and composition of the TFP are hugely significant because the cost of the TFP is the basis for much of the federal food assistance, including benefit allotments under the Supplemental Nutrition Assistance Program or SNAP [1,21,22,23,24]. In the fiscal year 2021, the federal government spent about 111 billion USD on SNAP [25]. Of that, about 105 billion USD were spent on benefits that households use to buy food [24]. Increased SNAP benefits, following the TFP 2021 revision, were recently the topic of congressional hearings [26].

The present focus was on protein foods, which accounted for about 25% of the TFP 2021 total costs. Protein foods in the TFP 2021 were represented by meat (i.e., beef and pork combined); poultry; eggs; seafood; and nuts, seeds, and soy products. With some exceptions, meat is generally more expensive than poultry. Since the June 2021 prices were higher for meat than for poultry, the proportion of poultry in the TFP 2021 market baskets was higher compared to the amounts of other protein foods. Seafood was the most expensive, with a cost of USD 12.80/week for a reference family of four. The TFP 2021 limited selections within the seafood category to lower-cost items such as tilapia or canned tuna.

Not all meat is equally expensive. Higher overall prices for the “meat” modeling category may have been due to the fact that the non-poultry meats included both beef and pork. The TFP 2021 considered numerous cuts of fresh pork: pork chop (baked, broiled, stewed, or fried), pork roast, and pork steak/cutlet as higher nutrient-dense meats for modeling purposes [2]. Multiple cuts of beef were also included. The present departure from the TFP 2021 was to separate the non-poultry meats into fresh pork and fresh beef. Given the disparity in prices, we specifically wanted to assess the place of fresh pork in the TFP 2021, and for that, we needed to separate pork from beef.

The TFP 2021 protocols [2] selected the most nutrient-dense foods from among lower-cost options within each food group. Our methods closely tracked those used by the USDA. We used the same publicly available input data and the same modeling categories. Foods and beverages reported in the What We Eat in America surveys were categorized based on price, nutrient density, and nutrient composition.

Our five models were used to generate healthy food plans for each of the eight age-gender groups, subject to nutrition, cost, and practicality constraints. Model 1 showed that were able to replicate the TFP 2021; the generated food plan price was the same as in the TFP 2021. The cost share by the food group was much the same as in the TFP 2021 [2].

Model 2 showed that the optimization model selected pork rather than beef or cured meat. Model 3 showed that the lowest cost healthy diet could be achieved with pork. Models 4 and 5 replaced all meat and poultry (but not fish) with either pork or beef. All food plans were adequate in nutrients, met the definition of healthy diets, and did not depart too far from observed eating habits. The main differences were in the estimated weekly cost; the pork-only diet was much lower in cost than the beef-only diet.

By law, the USDA must reevaluate the TFP every five years [7]. This first reevaluation was an iterative process conducted by economists, nutrition scientists, and analysts at the USDA CNPP in consultation with internal and external stakeholders. While the process has come under some scrutiny [27], MS-Nutrition, an external party based in France, relied on published and disclosed CNPP documentation to replicate the main TFP 2021 protocols. We used publicly available USDA databases and supplemental data on modeling categories and food prices, all published online. The data inputs, constraints, technical assumptions, and optimization models were publicly available. We also benefitted from direct advice and consultations with CNPP leadership and technical staff.

However, our analyses had some weaknesses. When it came to food amounts (rather than prices), there was one point of difference between the TFP 2021 and our models. We did follow the TFP 2021 in converting the modeling categories into market basket categories, following the same USDA aggregation codes. Market basket categories correspond to the food groups and subgroups featured in the Dietary Guidelines. However, the TFP 2021 went a step further using foods as consumed to calculate the amounts of foods purchased. That requires reverse adjusting for yield, preparation, and loss (adding peels, stems, bones, etc.) [28]. We aggregated foods into the same categories but stayed with foods as consumed (not purchased). There are differences in weight because of preparation and loss, especially for vegetables, but there was no effect on food plan cost. For example, for males 20–50, the “as-consumed” price was USD 59.78/week vs. USD 59.65 for the “as-purchased” price as shown in TFP 2021. Finally, there are a number of alternative optimization algorithms [29].

## 5. Conclusions

Our optimization analyses went beyond the TFP 2021 by separating non-poultry meats into pork and beef. First, our version of the TFP 2021 showed that the optimization model preferentially selected pork to arrive at the lowest-cost healthy diets that met all nutrient requirements, followed dietary guidance, and respected existing eating habits. Subsequent models showed that healthy food plans on a budget could be generated using pork as the only source of non-poultry meat or as the only source of all meat, other than fish. While all five food plans met nutrition and practicality criteria, fresh pork had the price advantage.

The planned regular updates of the TFP will provide an opportunity to tailor practical, healthy, and budget-conscious diets to individuals and households at nutrition risk [30]. In the future, we may want to show how a variety of nutrient-dense, lower-price options can support a healthy diet aligned with personal preferences and cultural foodways for population subgroups. Regardless, the food plans developed by optimization analyses [12] will inform research, education, and policy.

## Figures and Tables

**Table 1:** Characteristics of the five quadratic programming (QP) optimization models.

	Model 1 USDA TFP	Model 2 MS TFP	Model 3 Lowest Cost	Model 4 Pork Only	Model 5 Beef Only
Protein foods					
Beef/pork	Pooled	Separate	Separate	Separate	Separate
Beef	Amount of red meat defined by QP	Same amount of red meat as in Model 1	Defined by QP	Set to 0	Defined by QP
Pork			Defined by QP	Defined by QP	Set to 0
Cured meat	Defined by QP	Defined by QP	Defined by QP	Defined by QP	Defined by QP
Poultry	Defined by QP	Defined by QP	Defined by QP	Set to 0	Set to 0
Eggs, seafood, nuts, and soy	Defined by QP				
**Constraints**					
Total cost	Search for lowest cost	Set to the cost of Model 1	Search for lowest cost	Search for lowest cost	Search for lowest cost
Nutrients	Min/max amounts from DRIS for each age-gender group				
Food groups	Min/max amounts from healthy dietary patterns for each age-gender group				
**Objective function**	Deviation from observed intakes weighted by food prices—minimized				

**Table 2:** Quantity as consumed (lbs/wk) and cost (USD/wk) by food group for a family of four.

Market Basket Categories	Model 1		Model 2		Model 3		Model 4		Model 5	
	Quantity, lbs	Cost, USD	Quantity, lbs	Cost, USD	Quantity, lbs	Cost, USD	Quantity, lbs	Cost, USD	Quantity, lbs	Cost, USD
Vegetables	24.73	41.31	24.16	40.20	24.68	41.27	24.28	40.71	24.95	42.36
Fruits	22.79	27.62	23.66	27.72	22.71	27.46	22.47	27.54	20.90	26.45
Grains	18.74	29.06	19.02	28.20	18.21	28.33	17.21	27.24	18.14	31.66
Dairy	41.57	27.54	36.11	29.11	41.41	27.65	41.74	27.21	43.43	26.74
Protein foods	14.18	48.13	14.33	46.68	14.46	47.42	14.09	47.53	14.00	77.73
Miscellaneous	16.35	16.22	17.30	17.96	16.33	15.68	17.33	18.34	16.33	15.64
Total	138.36	189.88	134.59	189.87	137.81	187.81	137.11	188.57	137.75	220.58

**Table 3:** Quantity as consumed (lbs/wk) and cost (USD/wk) by protein food group for a family of four.

Protein Food Market Basket Categories	Model 1		Model 2		Model 3		Model 4		Model 5	
	Quantity, lbs	Cost, USD	Quantity, lbs	Cost, USD	Quantity, lbs	Cost, USD	Quantity, lbs	Cost, USD	Quantity, lbs	Cost, USD
Beef	2.05 *	9.00	0.00	0	0.00	0	0.00	0	7.92	56.11
Pork			2.05	6.9	3.40	11.48	7.92	26.71	0.00	0
Cured meat	0	0	0.00	0	0.00	0	0.00	0	0.00	0
Poultry	5.37	17.27	5.67	18.26	4.52	14.54	0.00	0	0.00	0
Eggs	2.08	3.43	1.99	3.28	1.93	3.17	1.51	2.5	1.60	2.66
Seafood	2.72	13.38	2.72	13.38	2.72	13.38	2.72	13.38	2.72	13.38
Nuts, seeds, soy	1.97	5.05	1.89	4.86	1.89	4.85	1.93	4.94	1.75	5.58
All categories	14.18	48.13	14.33	46.68	14.46	47.42	14.09	47.53	14.00	77.73

* Red meat beef and pork together.

**Table 4:** Total food plan costs and costs for protein foods in USD/week by age and gender group.

Age-Gender	USDA	Model 1	Model 2	Model 3	Model 4	Model 5
Market basket cost USD/week						
Female 4–13 y	--	40.21	40.25	39.70	39.83	47.09
Female 14–19 y	48.77	48.72	48.73	47.88	48.38	60.64
Female 20–50 y	47.86	47.81	47.80	47.39	47.86	56.29
Female 51–70 y	44.42	44.37	44.37	43.89	44.66	52.51
Male 4–13 y	--	42.08	42.05	41.58	41.73	49.50
Male 14–19 y	61.32	61.45	61.47	60.62	60.61	78.18
Male 20–50 y	59.65	59.78	59.77	59.14	59.15	67.70
Male 51–70 y	52.43	52.36	52.36	51.52	51.38	60.05
Protein foods market basket cost USD/week						
Female 4–13 y	--	10.30	9.76	10.40	10.39	16.93
Female 14–19 y	12.14	12.17	11.44	11.95	12.02	19.96
Female 20–50 y	12.03	12.40	11.87	12.00	12.00	19.71
Female 51–70 y	11.53	11.70	11.22	11.51	11.55	17.91
Male 4–13 y	--	10.58	10.09	10.78	10.78	17.80
Male 14–19 y	15.38	15.04	14.47	18.01	17.69	31.45
Male 20–50 y	14.49	14.85	14.96	14.24	14.36	23.29
Male 51–70 y	14.27	14.72	14.54	14.40	14.45	23.18

## Data Availability

The datasets analyzed for this study can be found on Food Data Central and in the supplemental data files for the TFP 2021.

## Supplementary Materials

The following supporting information can be downloaded at: https://www.mdpi.com/article/10.3390/nu15081897/s1, Table S1: Categorization of Market Basket, combined, TFP and modeling categories and Table S2: Total cost and detailed cost by ‘as-consumed’ Market Basket category for protein foods in Models 1-5 by food group and age-gender group.
