# Supplementary material for: Fresh Pork as Protein Source in the USDA Thrifty Food Plan 2021: A Modeling Analysis of Lowest-Cost Healthy Diets
Source: Nutrients. 2023 Apr 14;15(8):1897. doi: 10.3390/nu15081897 (PMC10146423; doi:10.3390/nu15081897)
Supplement: Supplementary file 1 [file nutrients-15-01897-s001.zip › Table S2_Nutrients_Submitted.pdf]

Supplemental Table S2 : Total cost and detailed cost by 'as-consumed' Market Basket category for protein foods in Models 1-5 by food group and age-gender group.

**Age**	**Food group**	**FEMALE**					**MALE**				
		Model 1	Model 2	Model 3	Model 4	Model 5	Model 1	Model 2	Model 3	Model 4	Model 5
**4-13**	Protein foods	10.3	9.76	10.4	10.39	16.93	10.58	10.09	10.78	10.78	17.8
	Beef	2.05	0	0	0	12.75	1.78	0	0	0	13.36
	Pork		1.57	2.15	6.07	0		1.37	2.38	6.36	0
	Cured meat	0	0	0	0	0	0	0	0	0	0
	Poultry	3.73	3.69	3.74	0	0	4.16	4.11	3.8	0	0
	Eggs	0.64	0.62	0.63	0.44	0.21	0.63	0.61	0.61	0.42	0.23
	Seafood	3.06	3.06	3.06	3.06	3.06	3.17	3.17	3.17	3.17	3.17
	Nuts, seeds, soy	0.82	0.82	0.82	0.82	0.91	0.84	0.83	0.82	0.83	1.04
	All categories	40.25	40.25	39.69	39.83	47.11	42.07	42.07	41.58	41.72	49.49
**14-19**	Protein foods	12.17	11.44	11.95	12.02	19.96	15.04	14.47	18.01	17.69	31.45
	Beef	3.44	0	0	0	15.71	2.42	0	0	0	26.45
	Pork		2.64	3.54	7.48	0		1.89	7.26	12.59	0
	Cured meat	0	0	0	0	0	0	0	0	0	0
	Poultry	4.01	4	3.76	0	0	6.73	6.68	5.09	0	0
	Eggs	0.45	0.54	0.38	0.28	0.24	0.79	0.8	0.56	0.26	0.19
	Seafood	3.3	3.3	3.3	3.3	3.3	3.85	3.85	3.85	3.85	3.85
	Nuts, seeds, soy	0.97	0.96	0.97	0.96	0.71	1.25	1.25	1.25	0.99	0.96
	All categories	48.72	48.72	47.88	48.37	60.62	61.46	61.46	60.62	60.62	78.19
**20-50**	Protein foods	12.4	11.87	12	12	19.71	14.85	14.96	14.24	14.36	23.29
	Beef	1.95	0	0	0	13.66	3.22	0	0	0	16.34
	Pork		1.49	1.58	6.5	0		2.47	5.37	7.78	0
	Cured meat	0	0	0	0	0	0	0	0	0	0
	Poultry	4.7	4.65	4.7	0	0	4.68	5.81	2.3	0	0
	Eggs	0.99	0.97	0.96	0.75	1.03	1.17	1.08	0.97	0.89	1.19

	Seafood	3.3	3.3	3.3	3.3	3.3	3.85	3.85	3.85	3.85	3.85
	Nuts, seeds, soy	1.46	1.46	1.46	1.45	1.72	1.93	1.75	1.75	1.84	1.91
	All categories	47.81	47.81	47.39	47.88	56.28	59.78	59.78	59.15	59.15	67.69
**51-70**	Protein foods	11.7	11.22	11.51	11.55	17.91	14.72	14.54	14.4	14.45	23.18
	Beef	1.83	0	0	0	12.02	3.57	0	0	0	15.39
	Pork		1.41	1.6	5.7	0		2.74	5.57	7.33	0
	Cured meat	0	0	0	0	0	0	0	0	0	0
	Poultry	3.84	3.79	3.91	0	0	3.86	4.56	1.67	0	0
	Eggs	1.12	1.11	1.09	0.94	0.85	1.3	1.26	1.18	1.14	1.28
	Seafood	3.14	3.14	3.14	3.14	3.14	3.59	3.59	3.59	3.59	3.59
	Nuts, seeds, soy	1.77	1.77	1.77	1.77	1.9	2.4	2.39	2.39	2.39	2.92
	All categories	44.38	44.38	43.89	44.66	52.5	52.36	52.36	51.52	51.38	60.06
